# Analysis of pathogenesis and drug treatment of chronic obstructive pulmonary disease complicated with cardiovascular disease

**DOI:** 10.3389/fmed.2022.979959

**Published:** 2022-11-04

**Authors:** Xiao-Fang Li, Cheng-Quan Wan, Yi-Min Mao

**Affiliations:** ^1^Department of Respiratory Medicine, The First Affiliated Hospital, College of Clinical Medicine of Henan University of Science and Technology, Luoyang, Henan, China; ^2^Department of Neonatology, Luoyang Maternal and Child Health Hospital,, Luoyang, Henan, China

**Keywords:** COPD, CVD, pathophysiology, medical treatment, management

## Abstract

Chronic obstructive pulmonary disease (COPD) is a disease characterized by persistent airflow limitation, and is associated with abnormal inflammatory responses in the lungs to cigarette smoke and toxic and harmful gases. Due to the existence of common risk factors, COPD is prone to multiple complications, among which cardiovascular disease (CVD) is the most common. It is currently established that cardiovascular comorbidities increase the risk of exacerbations and mortality from COPD. COPD is also an independent risk factor for CVD, and its specific mechanism is still unclear, which may be related to chronic systemic inflammation, oxidative stress, and vascular dysfunction. There is evidence that chronic inflammation of the airways can lead to destruction of the lung parenchyma and decreased lung function. Inflammatory cells in the airways also generate reactive oxygen species in the lungs, and reactive oxygen species further promote lung inflammation through signal transduction and other pathways. Inflammatory mediators circulate from the lungs to the whole body, causing intravascular dysfunction, promoting the formation and rupture of atherosclerotic plaques, and ultimately leading to the occurrence and development of CVD. This article reviews the pathophysiological mechanisms of COPD complicated by CVD and the effects of common cardiovascular drugs on COPD.

## Introduction

Chronic obstructive pulmonary disease (COPD) is a chronic respiratory disease with persistent airflow limitation. The airflow limitation is usually gradual and not fully reversible. It is mainly related to smoking and inhalation of other toxic and harmful gases (1). COPD is the fourth leading cause of death in the world, and the number of deaths is increasing year by year (2, 3). In addition, with the aging of the population and smoking, the economic and social burden of COPD is also increasing year by year (2, 4). Age and smoking are also high-risk factors for many other diseases, making COPD patients prone to multiple other comorbidities, which complicates treatment (5). Comorbidities are prevalent in COPD patients, with approximately 86–98% of COPD patients having at least one comorbidity (6). The presence of comorbidities reduces patients’ quality of life and increases in-hospital mortality, and many patients with mild to moderate COPD die from comorbidities rather than COPD itself (7). The current treatment of COPD mainly includes two aspects, one is conventional treatment, including smoking cessation, bronchodilator, pulmonary rehabilitation and vaccination, and the other is the management of comorbidities (7, 8). The management of comorbidities is related to the quality of life of patients with early COPD (9), and reasonable treatment can reduce the mortality and economic burden of end-stage patients (2).

Cardiovascular disease is one of the most common comorbidities of COPD (10), which is related to the morbidity and mortality of COPD patients, and is associated with various diseases, such as acute myocardial infarction, arrhythmia, heart failure, etc. (11). A recent study by Pikoula et al. showed that COPD patients with CVD had an increased risk of COPD exacerbations and were most likely to die from circulatory disease, suggesting that CVD has an adverse effect on the COPD population (12). COPD and CVD frequently occur in the same individual, and the presence of overlapping symptoms (chest tightness, dyspnea) makes treatment and management of the disease often incomplete (13). Nearly half of the hospitalizations and deaths in COPD patients are attributed to CVD (14). A comprehensive treatment is required for such patients. Drugs for the treatment of CVD have both benefits and possible side effects for COPD. This article reviews the pathophysiological mechanisms of COPD complicated by CVD and the effects of common cardiovascular drugs on COPD.

## Common pathophysiological mechanism of chronic obstructive pulmonary disease and cardiovascular disease

### Inflammation and oxidative stress

Chronic systemic inflammation plays an important role in the pathogenesis of CVD (15, 16) and is involved in the occurrence, development and rupture of atherosclerotic plaques (17), leading to coronary heart disease and heart failure (18). During inflammation, monocytes migrate from the blood into and under the intima, phagocytose other cells and toxic molecules (such as oxidized low-density lipoprotein [oxLDL]), produce inflammatory cytokines, and can differentiate into inflammatory dendrites shape cells, macrophages, or foam cells, forming early plaques. Early plaques mature into atherosclerotic plaques as inflammatory cells and lipids accumulate. Over time, matrix-degrading proteases and cytokines secreted by macrophages can lead to the breakdown of the outer fibrous cap of atheromatous plaques. The expelled plaque fragments release tissue factor into the blood, triggering a coagulation cascade and thrombosis, leading to acute narrowing of the arteries, leading to acute coronary syndrome, myocardial infarction (19). This suggests that inflammation is involved in the entire process of atherosclerosis, from the onset of injury to the onset of clinical symptoms. The levels of circulating inflammatory markers, such as intercellular cell adhesion molecule 1 (ICAM-1), interleukin-6, C-reactive protein, and serum amyloid-A, are also associated with cardiovascular disease prognosis (16). Other studies have shown that inflammation is also involved in the occurrence of arrhythmia. During systemic inflammation, the incidence of atrial fibrillation increases, especially in sepsis, and plasma C-reactive protein (CRP) levels increase before the onset of atrial fibrillation. Infiltration of CD68-positive macrophages and increased expression of IL-6 and transforming growth factor beta (TGFβ), inflammatory factors mainly derived from epicardial adipose tissue, were seen in atrial tissue from patients with atrial fibrillation (AF). Under the induction of systemic inflammatory response, epicardial adipose tissue increases and secretes pro-inflammatory cytokines. These inflammatory factors act on atrial cardiomyocytes, reduce cardiomyocyte conduction velocity, shorten action potential duration (APD), and ultimately lead to atrial arrhythmias happened (20). The inflammasome may be another mechanism by which inflammation induces arrhythmias. The inflammasome, a multi-protein complex assembled by cytoplasmic pattern recognition receptors (PRRs), is an important part of the innate immune system and can recruit and activate the proinflammatory protease caspase-1 under pathological conditions. Activated caspase-1 cleaves the precursors of IL-1β and IL-18 to produce the corresponding mature cytokines. Cholesterol-rich Western diet triggers NLRP3 inflammasome-dependent innate immune rearrangement, exerting inflammation-related arrhythmias (21). Inflammation is a hallmark feature of COPD and one of the mechanisms that affects distant tissues and organs and increases the prevalence of CVD (22). COPD is considered an inflammatory disease with infiltration of various inflammatory cells including neutrophils, mast cells, eosinophils, CD8+ T lymphocytes, and macrophages in the airways (23, 24). Smoking is a common risk factor for COPD and CVD. Smoking can cause various inflammatory responses in susceptible people. COPD is thought to be the result of an enhanced lung inflammatory response to noxious gases such as cigarette smoke and other noxious particles, leading to airflow limitation that is not fully reversible (2, 25). In COPD patients, blood inflammatory markers such as CRP were elevated, while patients with CVD had higher blood concentrations of fibrinogen, interleukin-6, interleukin-8, and other inflammatory markers than patients without comorbidities (26). Inflammatory response may be a key factor in the comorbidity of COPD and CVD, and it has even been suggested that COPD is only one of the manifestations of systemic inflammatory response (27). There is a markedly increased risk of CVD events within 30 days after COPD hospitalization and exacerbations (28), which is associated with increased circulating proinflammatory markers (29). Therefore, the inflammatory response state of COPD patients can promote the occurrence of CVD.

In addition to inflammation, oxidative stress is another feature of COPD that plays an important role in the development of the disease and is also associated with CVD (30). Oxidative stress (OS) mainly refers to a state of imbalance between oxidation and antioxidant effects in the body. OS is almost universal in cardiovascular diseases and is involved in myocardial ischemia-reperfusion injury (31, 32), heart failure (33, 34, 35), atherosclerosis (36, 37), atrial fibrillation (38–40) and hypertension (41–43), etc. An important link between oxidative stress and cardiovascular outcomes has been established, supported by extensive clinical trial data. A study of 636 people showed that levels of the antioxidant enzyme glutathione peroxidase-1 were positively associated with the incidence of cardiovascular events (44). The reactive oxygen species in the heart mainly come from NADPH oxidase, mitochondria, xanthine oxidase and unconjugated nitric oxide synthase (NOS). In patients with coronary heart disease and heart failure, the electron transport chain of mitochondria is disturbed, and the expression and activity of NADPH oxidase and xanthine oxidase are increased. NOS uncoupling and structural instability lead to increased ROS production. Excessive ROS causes cellular dysfunction, protein and lipid peroxidation, DNA damage, and ultimately irreversible cellular damage and apoptosis (45, 46). Under oxidative stress, ROS can lead to uncoupling of NOS by disrupting the vascular protective NO signaling pathway. Thereby mediating endothelial dysfunction and vascular abnormalities. When nitric oxide synthase is uncoupled, nitric oxide is converted to superoxide anion 2-) and pernitrite (ONOO-), resulting in reduced NO bioavailability and vasoconstriction. In addition, pernitrite can inhibit vasodilation, oxidize DNA and lipids, and reduce the inhibition of platelet aggregation by NO, thus participating in the progression of atherosclerosis, and cardiovascular diseases are mostly caused by atherosclerosis (47). At the same time, increasing evidence also indicates that oxygen species has a potential role in the pathophysiology of AF and ventricular arrhythmia. Reactive oxygen species may mediate the formation of ectopic heart rhythms by altering the homeostasis of Ca2+, K+, and Na+ channels on the cardiomyocyte membrane and causing gap junction remodeling, but the exact molecular mechanism remains to be determined (48, 49). Cigarette smoke is the main source of oxidants in the lungs, and inflammatory cells in the airways also produce reactive oxygen species (ROS) in the lungs (50). The production of reactive oxygen species in COPD patients results from increased nicotinamide adenine dinucleotide phosphate (NADPH) activity in macrophages, neutrophils, and epithelial cells (14). Compared with non-smokers, the proportion of macrophages and neutrophils in the lung tissue of smokers and COPD patients increased, and the oxidative stress in the lungs of COPD patients was further aggravated by activating NADPH to generate reactive oxygen species (51). These reactive oxygen species enhance pulmonary inflammatory responses by activating the transcription factor nuclear factor kappa B, mitogen-activated protein kinase (MAPK) signaling, chromatin remodeling, and pro-inflammatory gene transcription (51).

Cardiovascular events are one of the leading causes of hospitalization in patients with COPD and contribute significantly to the cost burden of the disease. One study found that among smokers with mild to moderate COPD, CVD accounted for 50% of hospitalizations (52). Inflammation and oxidative stress associated with COPD may be the mechanisms linking COPD with an increased risk of CVD.

### Vascular endothelial dysfunction

Vascular endothelium is widely distributed in the body and can maintain the tension of blood vessels and the structure of blood vessels by mediating vasodilation, contraction, production inhibition and production promotion, as well as the balance of anti-inflammatory and pro-inflammatory, and can synthesize and release vasoactive substances., to regulate vascular tone, thereby regulating platelet function, inflammatory response, and vascular smooth muscle cell growth and migration (53). Vascular endothelial dysfunction refers to the imbalance between vasodilator and vasoconstrictor factors produced by endothelial cells, which is the main factor of atherosclerosis and occurs in the early stages of atherosclerosis (54, 55). Atherosclerosis is a major factor in the pathogenesis of CVD (56). The increased risk of acute atherothrombotic events in COPD is independent of smoking and other cardiovascular risk factors. Airway inflammation in COPD spreads to the systemic circulation and plays a key role in plaque formation and rupture (57). The most important vasodilatory substance released by the vascular endothelium is nitric oxide (NO), which can inhibit growth and inflammation, and has anti-platelet aggregation effects. Decreased NO is a manifestation of endothelial dysfunction and is associated with decreased NO synthase activity (competitive inhibition by L-arginine or competitive inhibition by asymmetric dimethylarginine) or decreased NO bioavailability (overexpression of endothelin-1) (58–60). Circulating concentrations of asymmetric dimethylarginine (ADMA) are significantly elevated in COPD patients and are significantly correlated with disease progression. Elevated ADMA inhibits NOS activity and further increases endothelial dysfunction (53). It has been found that NO-mediated endothelial relaxation in COPD patients is present early in the disease and is associated with airflow obstruction (61). The results of multiple meta-analyses have also confirmed that COPD endothelial function is impaired, and the severity is positively correlated with the degree of airway obstruction (62–64). Endothelial dysfunction in COPD may be involved in the following aspects (65): (1) Toxic effects of cigarette smoke; (2) Production of autoantibodies in endothelial cells; (3) Vascular inflammation; (4) Increased levels of oxidative stress; (5) Antioxidant Pathway activation decreased; (6) NO release decreased, endothelin-1 expression increased and so on. Insulin resistance may also be involved in COPD vascular dysfunction (66). When insulin resistance occurs, insulin signal transduction changes, eNOS activity is down-regulated, and elevated blood sugar can lead to increased glycation end products, which promote vascular inflammation, inhibit NO production and release, and lead to endothelial dysfunction.

Vascular endothelial injury in COPD patients mediates the occurrence of CVD, which is the result of enhanced oxidative stress and inflammatory response, thereby significantly increasing the risk of vascular diseases such as atherosclerosis, myocardial infarction, and stroke (67). In the initial stage of atherosclerotic injury, the damage of vascular endothelial function can be seen. Under the stimulation of inflammation, vascular endothelial cells secrete vascular cell adhesion molecule-1 (VCAM-1), which promotes the adhesion and aggregation of inflammatory cells, thereby exerting inflammatory damage (68). Studies have shown that when inflammatory mediators are removed, the integrity of the vascular endothelium can be restored, and the incidence of CVD will be reduced (69). Inflammatory factors can enhance NOX-NADPH oxidase activity resulting in increased ROS production, which induces vessel wall inflammation through activation of nuclear factor κ-light chain enhancer (NFκB) signaling in B cells (70). In addition, increased ROS production can lead to the rapid inactivation of NO to form peroxynitrite, a strong oxidant molecule that simultaneously uncouples endothelial nitric oxide synthase (eNOS), resulting in increased superoxide and activity NO production is further reduced, ultimately promoting the formation of atherosclerosis (71).

### Hypoxia

Hypoxia refers to a pathological process in which the metabolism, function and morphological structure of tissues are abnormally changed due to insufficient oxygen supply or oxygen use disorders in tissues. Hypoxia is a common clinical pathological process, and hypoxia of important organs such as brain, heart and lungs is also an important cause of death. There is evidence that hypoxia plays a detrimental role in CVD (72). Hypoxia enhances the expression of hypoxia-inducible genes, which are related to vascular endothelial production, erythropoiesis, cell metabolism, and inflammatory processes, and participate in atherosclerosis, vascular and cardiac remodeling by altering cell transduction pathways (73). The maintenance of normal vascular function depends on the production and regulation of NO. NO synthase is responsible for the production of endothelial NO. Hypoxia can cause post-translational modification of NO synthase, leading to the disturbance of NO production, ultimately causing vascular dysfunction and driving the occurrence of CVD and development (13). Long-term chronic hypoxia in COPD patients induces systemic inflammation, oxidative stress, foam cell production, and increased expression of cell adhesion molecules in endothelial cells, leading to the progression of atherosclerosis, which in turn induces CVD (74). Long-term chronic hypoxia in COPD patients is also a key driver of pulmonary hypertension. Hypoxia-induced vasoconstriction, systemic inflammation, endothelial dysfunction, and erythrocytosis, as well as persistent lung inflammation from increased ROS and reactive nitrogen species (RNS) *in vivo*, all promote pulmonary arterial remodeling, vessel narrowing, and subsequent increased pulmonary arterial pressure. Later, it can lead to right heart enlargement, followed by pulmonary heart disease and ventricular failure (14, 27). The incidence of myocardial infarction and heart failure in these patients was significantly increased.

**FIGURE 1:**
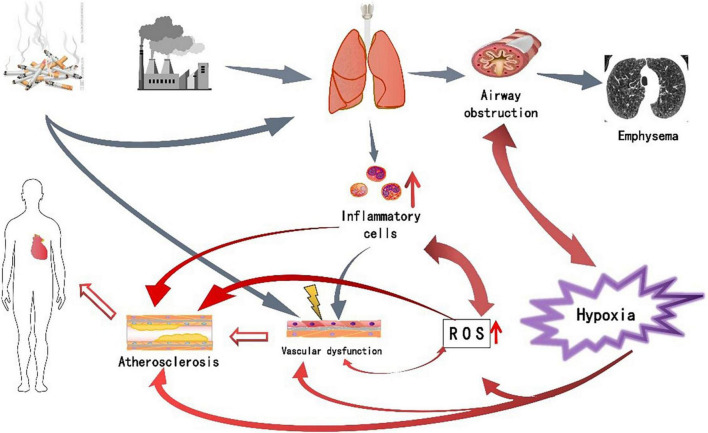
The pathogenesis of chronic obstructive pulmonary disease (COPD) complicated with cardiovascular disease (CVD) and the relationship between various factor.

Chronic obstructive pulmonary disease is an independent risk factor for CVD (75), and CVD is very prevalent in COPD patients and is associated with considerable morbidity and mortality, a condition that is increasingly seen across the “cardiopulmonary continuum”. In the context, both diseases are associated with systemic inflammation, with the involvement of hypoxia, oxidative stress and other factors (76). The above-mentioned multiple pathogenic mechanisms do not act independently, but are interrelated and promote each other. The persistent respiratory symptoms of COPD are caused by abnormalities in the airways and alveoli that result from chronic lung inflammation, and oxidative stress exacerbates the inflammatory response in the airways, while increased oxidative stress leads to endothelial dysfunction. Deficiency of vascular endothelial antioxidant factor NO increases oxidative stress. In COPD, hypoxia increases oxidative stress and the production of reactive oxygen species, while reducing the production of vascular endothelial antioxidant factors. A vicious cycle is formed between these factors, which eventually leads to the occurrence of CVD (Figure 1). Understanding these pathophysiological mechanisms could provide new directions for disease treatment. Since oxidative stress is a key factor in disease progression, drugs against oxidative stress have attracted more and more attention in recent years. Animal studies have shown that Apocynin, an inhibitor of reactive oxygen species generation, can reduce cigarette smoke-induced lung inflammation in mice (77). Funamoto’s research shows that curcumin can reduce oxidized low-density lipoprotein, thereby delaying the occurrence and development of atherosclerosis, which may be related to curcumin’s inhibition of NF-κB to produce antioxidant activity, inhibit inflammation and oxidative stress reaction (78). Therefore, antioxidant therapy may become a new target for the treatment of COPD combined with CVD.

### Effects of common therapeutic drugs for cardiovascular disease on chronic obstructive pulmonary disease

Chronic obstructive pulmonary disease and CVD are closely related. Due to overlapping symptoms and signs, clinical symptoms are often attributed to one disease, while another disease is ignored. In patients with newly diagnosed CVD, uncertainty about the safety of some physicians has also led to underuse of CVD drugs, even in patients with known CVD (8). The use of cardiovascular drugs may be beneficial to patients, reducing the risk of disease progression and death to a certain extent (79, 80).

### Beta-blockers

With the aging of the population, the increase in air pollution and smoking rates, the global economic burden of COPD is increasing, and most COPD patients are complicated by CVD, which prolongs the hospitalization period, costs a lot and has a poor prognosis. Beta-agonists can stimulate beta-receptors on airway smooth muscle to produce bronchodilator effects and are the basis for COPD, while beta-blockers can counteract the toxicity of catecholamine adrenergic transmitters, especially through beta1 receptors mediated cardiotoxicity, as well as anti-hypertension, anti-myocardial ischemia, blocking the renin-angiotensin-aldosterone system by inhibiting renin release, improving cardiac function and increasing left ventricular ejection fraction, antiarrhythmic and other effects, is the standard treatment of many CVD drugs. The use of beta-blockers in COPD patients has been controversial because of their opposing pharmacological effects. The 2016 European Society of Cardiology guidelines recommend the use of beta-blockers in patients with COPD and CVD (81). Beta-blockers are underused in COPD patients with CVD due to concerns about potential side effects, resulting in poor patient outcomes (82, 83).

Beta -adrenergic receptors are divided into three subtypes. β1 and β3 are mainly distributed on cardiomyocytes. After β1 receptor activation, it can have positive effects on the myocardium, and after β3 receptor activation, it can produce negative inotropic effect. β2 receptors are mainly distributed on smooth muscle, and the activation of this receptor can cause smooth muscle relaxation (84). Beta-blockers are classified into non-selective beta-blockers and cardioselective beta-blockers according to their receptor subtypes. Cardioselective β-receptor antagonists have higher selectivity for cardiac β1-receptors, and the adverse reactions such as bronchospasm are milder at the treatment dose. A growing number of clinical trials and meta-analyses have also shown that the use of selective beta-blockers in COPD patients is safe and reduces all-cause and in-hospital mortality. Studies have shown that beta-blockers do not exacerbate COPD, and even suggest that cardioselective beta-blockers are beneficial for COPD. A study on the efficacy of bisoprolol in the treatment of COPD complicated with heart failure showed that the bisoprolol treatment group could effectively improve blood gas indexes, improve left ventricular systolic and diastolic function, improve quality of life, and relieve clinical symptoms (85). In a study of patients with moderate to severe COPD, there was no significant difference in the risk of exacerbation of COPD between the metoprolol group and the placebo group (86). A recent study on the effect of beta-blockers on the long-term prognosis of Asian COPD patients with heart failure showed that beta-blockers can reduce all-cause mortality in these patients. At the same time, active use of beta-blockers in such patients is advocated (87). Results of a recent large meta-analysis showed that beta-blockers are safe and reduce all-cause and in-hospital mortality in patients with COPD, and selective beta-blockers may even reduce the acute incidence of COPD. It does not affect the effect of bronchodilators and can resist the increased heart rate caused by bronchodilators. Therefore, the use of beta-blockers should not be restricted in patients with COPD and heart disease (88).

At present, most of the data are derived from observational studies or based on retrospective studies. The sample size is small and the observation time is short, and there may be some bias. More high-quality RCT studies are still needed to further demonstrate the safety of β-blockers in patients with COPD.

### Statins

Statins, i.e., 3-hydroxy-3-methylglutaryl coenzyme A (HMG-CoA) reductase inhibitors, potently lower not only total cholesterol and LDL but also triglycerides to a certain extent, can also increase high-density lipoprotein, so statins are more comprehensive lipid-lowering drugs (89). The mechanism of action of statins is to competitively inhibit the endogenous cholesterol synthesis rate-limiting enzyme HMG-CoA reductase, block the intracellular oxyvalonate metabolic pathway, and reduce intracellular cholesterol synthesis. It is mainly used to treat atherosclerosis clinically, and it has become a commonly used drug for the prevention and treatment of coronary heart disease. In recent years, studies have found that statins have multiple non-lipid-lowering effects, including stabilizing atherosclerotic plaques, improving endothelial function, and anti-inflammatory and stabilizing lipid cores (90). Persistent airflow limitation in COPD is associated with chronic airway inflammation, and the anti-inflammatory and antioxidant effects of statins may reduce the risk of exacerbation in COPD patients (91, 92). Randomized controlled studies have shown that rosuvastatin can benefit patients with stable COPD by reducing systemic inflammation and improving endothelial-dependent vascular function *in vivo* (93). A later observational study also showed that COPD patients with cardiovascular indications and systemic inflammation may derive clinical benefit from statin therapy (94). The results of the STATCOPE study showed that simvastatin did not increase the number and duration of exacerbations in patients with moderate to severe COPD (95). However, the study data did not show the therapeutic benefit of statins in patients with moderate to severe COPD, and the benefit of statins in patients with COPD and CVD cannot be ruled out because patients with coronary heart disease were not included. A recent cohort observational study on the effect of statins on the acute exacerbation rate in COPD patients obtained similar results from the STATCOPE study, that is, statins had no effect on the exacerbation rate and mortality in COPD patients (96). The results of a recent systematic review evaluating the benefits and harms of statins compared with placebo in COPD patients showed that statin use can reduce CRP and IL-6 levels, but there were no significant gains in COPD mortality, exacerbation frequency, or lung function (97). Of course, the use of statins did not increase adverse effects (97). Several retrospective studies have shown that statins can reduce the risk of acute exacerbations in patients with COPD (98–101). The results of a recent study on the effect of losartan on the progress of emphysema have not been published yet (102). Statins also reduce the risk of pulmonary hypertension in COPD, with higher daily doses and longer lasting benefits (103).

### Renin-angiotensin-aldosterone system inhibitors

The renin-angiotensin-aldosterone system (RAAS) is a blood pressure regulating system produced by the kidneys in the body, causing vascular smooth muscle contraction and water and sodium retention, resulting in a blood pressure boosting effect. Renin-angiotensin-aldosterone system inhibitors, including angiotensin-converting enzyme inhibitors (ACEI) and angiotensin II receptor blockers (ARBs), are currently widely used in the prevention and treatment of cardiovascular diseases, while RAAS may promote the progression of COPD and pulmonary fibrosis by inducing the production and release of inflammatory factors and reactive oxygen species (104). There are relatively few data on the application of RAAS inhibitors in patients with COPD. A recent randomized controlled study showed that the use of RAAS inhibitors in patients with heart failure with COPD compared with those without COPD were consistent, meaning that RAAS inhibitors did not increase adverse outcomes in COPD patients (105). Angiotensin II receptor blockers are well tolerated in patients with stage III and IV COPD, and there may be potential benefit in patients with COPD and cardiovascular comorbidities (106). A Multi-Ethnic Study of Atherosclerosis Lung Study found that the use of ACEI or ARB can delay the development of emphysema, especially in smokers, and the efficacy is dose-related (107). The most common side effect of ACEIs is cough, usually dry or irritating, occurring in approximately 5–20% of patients (108). Studies have also shown that the use of ARBS drugs in COPD patients has a lower risk of exacerbation, pneumonia, and mortality compared with ACEI drugs (109). So angiotensin II antagonists may be a better choice when treatment is needed.

### Antiplatelet therapy

Thrombosis is one of the main causes of cardiovascular diseases. The adhesion, release and aggregation of platelets in blood vessels are the main causes of thrombosis. Therefore, antiplatelet therapy is widely used in some vascular diseases. Platelet activation is increased in COPD patients (110), it may be related to factors such as inflammatory response, hypoxia, and hemodynamic changes, which further increase the cardiovascular risk in COPD patients (111). In a study in patients with COPD exacerbations, thrombocytosis was associated with increased in-hospital and 1-year mortality, and antiplatelet therapy significantly reduced 1-year mortality (110). Results of a recent randomized controlled study showed that platelet response to aspirin and ticagrelor antiplatelet therapy was not observed in nearly one-third of COPD patients with no prior CVD history, supporting a high prothrombotic environment in COPD patients, suggesting that in addition to antiplatelet therapy, anticoagulation therapy may have an impact on CVD morbidity and mortality in COPD patients (112). The results of a meta-analysis demonstrated that antiplatelet therapy reduced all-cause mortality in COPD patients (113). A prospective multicenter study showed that antiplatelet drugs improved survival in COPD patients, possibly related to systemic antithrombotic effects (114). However, there are no studies showing that the use of aspirin can improve lung function in COPD patients (115). The results of another study showed that antiplatelet therapy significantly reduced the risk of ischemic events in patients with acute coronary syndrome and COPD, without increasing the total serious bleeding events (116).

### Diuretics

Diuretics, mainly divided into loop diuretics, thiazide diuretics, and potassium-sparing diuretics, are important components of the treatment strategy for patients with heart failure. The use of thiazide diuretics in COPD patients with hypertension has not been found to affect respiratory function and does not increase the risk of acute exacerbations in COPD patients. Thiazide diuretics are not contraindicated in COPD patients (117). There are few data on the use of loop diuretics in COPD patients. A recent study on diuretic use and adverse respiratory events in elderly COPD patients found that the proportion of patients receiving loop diuretics due to acute exacerbation or pneumonia was higher than the control group. While the rate of exacerbation was decreased in patients receiving thiazide diuretics, patients receiving potassium-sparing diuretics and carbonic anhydrase inhibitors did not differ significantly from controls (118). However, further research is needed to clarify whether the results come from causality or confounding factors. On the other hand, the use of diuretics can reduce pulmonary congestion and edema, increase lung compliance and improve pulmonary ventilation function (119, 120), which is beneficial for COPD patients. However, the related side effects caused by diuretics still need attention, such as hypokalemia, hypercapnia, metabolic alkalosis, and decreased cardiac output (121–123). Secondly, the excessive dosage of diuretics can lead to thick airway secretions in patients with COPD, thus making it difficult to expectoration and aggravating the disease. Therefore, the type and dosage of diuretics should be determined according to the condition, so as to achieve individualized treatment.

By reviewing the current literature, in general, most CVD drugs are relatively safe in COPD patients, and active drug intervention is encouraged for COPD patients with CVD.

## Conclusion

The comorbidities of COPD are very common and may even be fatal, and such patients should be actively intervened and managed. CVD is a common complication of COPD, among which atrial fibrillation, heart failure and ischemic cardiomyopathy are the most common, and many factors such as inflammation, oxidative stress, hypoxia, and vascular endothelial dysfunction are involved in this process. Due to overlapping symptoms and signs such as dyspnea and fatigue, clinical treatment is often insufficient, and acute respiratory symptoms are often caused by confounding factors in the lungs and heart, and most cardiovascular drugs do not increase the risk of COPD exacerbation, or even can improve prognosis. Therefore, comprehensive management and individualized treatment should be done for COPD patients with CVD.

## Author contributions

X-FL and C-QW designed the study. X-FL searched the literature and wrote the manuscript. Y-MM revised the manuscript. All authors contributed to the article and approved the submitted version.

## Abbreviations

COPD: chronic obstructive pulmonary disease
CVD: cardiovascular disease
ROS: reactive oxygen species
NADPH: nicotinamide adenine dinucleotide phosphate
MAPK: mitogen-activated protein kinase
NO: nitric oxide
ADMA: asymmetric dimethylarginine
VCAM-1: vascular cell adhesion molecule-1
eNOS: endothelial nitric oxide synthase
RAAS: renin-angiotensin-aldosterone system
ACEI: angiotensin-converting enzyme inhibitors
ARBs: angiotensin II receptor blockers
CRP: C-reactive protein
ICAM-1: intercellular cell adhesion molecule 1
AF: atrial fibrillation
PRRs: pattern recognition receptors
OS: Oxidative stress
RNS: reactive nitrogen species.

## References

[1] Labaki WW Rosenberg SR. Chronic obstructive pulmonary disease. *Ann Intern Med.* (2020) 173:ITC17–32. 10.7326/AITC202008040 32745458

[2] Global Initiative for Chronic Obstructive Lung Disease,. Global Strategy for the Diagnosis, Management, and Prevention of Chronic Obstructive Pulmonary Disease 2021 Report. Edinburgh: Global Initiative for Chronic Obstructive Lung Disease—GOLD (2021).

[3] Szalontai K Gémes N Furák J Varga T Neuperger P Balog JÁ Chronic obstructive pulmonary disease: epidemiology, biomarkers, and paving the way to lung cancer. *J Clin Med.* (2021) 10:2889. 10.3390/jcm10132889 34209651 PMC8268950

[4] Halpin DMG Celli BR Criner GJ Frith P López Varela MV Salvi S The GOLD summit on chronic obstructive pulmonary disease in low- and middle-income countries. *Int J Tuberc Lung Dis.* (2019) 23:1131–41. 10.5588/ijtld.19.0397 31718748

[5] Chatila WM Thomashow BM Minai OA Criner GJ Make BJ. Comorbidities in chronic obstructive pulmonary disease. *Proc Am Thorac Soc.* (2008) 5:549–55. 10.1513/pats.200709-148ET 18453370 PMC2645334

[6] Putcha N Drummond MB Wise RA Hansel NN. Comorbidities and chronic obstructive pulmonary disease: prevalence, influence on outcomes, and management. *Semin Respir Crit Care Med.* (2015) 36:575–91. 10.1055/s-0035-1556063 26238643 PMC5004772

[7] Recio Iglesias J Díez-Manglano J López García F Díaz Peromingo JA Almagro P Varela Aguilar JM. Management of the COPD patient with comorbidities: an experts recommendation document. *Int J Chron Obstruct Pulmon Dis.* (2020) 15:1015–37. 10.2147/COPD.S242009 32440113 PMC7217705

[8] Matsunaga K Harada M Suizu J Oishi K Asami-Noyama M Hirano T. Comorbid Conditions in chronic obstructive pulmonary disease: potential therapeutic targets for unmet needs. *J Clin Med.* (2020) 9:3078. 10.3390/jcm9103078 32987778 PMC7598716

[9] Koskela J Kilpeläinen M Kupiainen H Mazur W Sintonen H Boezen M Co-morbidities are the key nominators of the health related quality of life in mild and moderate COPD. *BMC Pulm Med.* (2014) 14:102. 10.1186/1471-2466-14-102 24946786 PMC4229911

[10] Magnussen H Disse B Rodriguez-Roisin R Kirsten A Watz H Tetzlaff K Withdrawal of inhaled glucocorticoids and exacerbations of COPD. *N Engl J Med.* (2014) 371:1285–94. 10.1056/NEJMoa1407154 25196117

[11] Müllerova H Agusti A Erqou S Mapel DW. Cardiovascular comorbidity in COPD systematic literature review. *Chest.* (2013) 144:1163–78. 10.1378/chest.12-2847 23722528

[12] Pikoula M Quint JK Nissen F Hemingway H Smeeth L Denaxas S. Identifying clinically important COPD sub-types using data-driven approaches in primary care population based electronic health records. *BMC Med Inform Decis Mak.* (2019) 19:86. 10.1186/s12911-019-0805-0 30999919 PMC6472089

[13] Deshmukh K Khanna A. Implications of managing chronic obstructive pulmonary disease in cardiovascular diseases. *Tuberc Respir Dis.* (2021) 84:35–45. 10.4046/trd.2020.0088 33045814 PMC7801809

[14] Brassington K Selemidis S Bozinovski S Vlahos R. New frontiers in the treatment of comorbid cardiovascular disease in chronic obstructive pulmonary disease. *Clin Sci.* (2019) 133:885–904. 10.1042/CS20180316 30979844 PMC6465303

[15] Drakopoulou M Toutouzas K Michelongona A Tousoulis D Stefanadis C. Vulnerable plaque and inflammation: potential clinical strategies. *Curr Pharm Des.* (2011) 17:4190–209. 10.2174/138161211798764816 22204378

[16] Linden F Domschke G Erbel C Akhavanpoor M Katus HA Gleissner CA. Inflammatory therapeutic targets in coronary atherosclerosis-from molecular biology to clinical application. *Front Physiol.* (2014) 5:455. 10.3389/fphys.2014.00455 25484870 PMC4240064

[17] Libby P. Inflammation in atherosclerosis. *Nature.* (2002) 420:868–74. 10.1038/nature01323 12490960

[18] Paulus WJ Tschope C. A novel paradigm for heart failure with preserved ejection fraction: comorbidities drive myocardial dysfunction and remodeling through coronary microvascular endothelial inflammation. *J Am Coll Cardiol.* (2013) 62:263–71. 10.1016/j.jacc.2013.02.092 23684677

[19] Woollard KJ Geissmann F. Monocytes in atherosclerosis: subsets and functions. *Nat Rev Cardiol.* (2010) 7:77–86. 10.1038/nrcardio.2009.228 20065951 PMC2813241

[20] Ihara K Sasano T. Role of inflammation in the pathogenesis of atrial fibrillation. *Front Physiol.* (2022) 13:862164. 10.3389/fphys.2022.862164 35492601 PMC9047861

[21] Andelova K Bacova BS Sykora M Hlivak P Barancik M Tribulova N. Mechanisms underlying antiarrhythmic properties of cardioprotective agents impacting inflammation and oxidative stress. *Int J Mol Sci.* (2022) 23:1416. 10.3390/ijms23031416 35163340 PMC8835881

[22] Almagro P Boixeda R Diez-Manglano J Gómez-Antúnez M López-García F Recio J. Insights into chronic obstructive pulmonary disease as critical risk factor for cardiovascular disease. *Int J Chron Obstruct Pulmon Dis.* (2020) 15:755–64. 10.2147/COPD.S238214 32341642 PMC7166051

[23] Mathioudakis AG Janssens W Sivapalan P Singanayagam A Dransfield MT Jensen JS Acute exacerbations of chronic obstructive pulmonary disease: in search of diagnostic biomarkers and treatable traits. *Thorax.* (2020) 75:520–7. 10.1136/thoraxjnl-2019-214484 32217784 PMC7279206

[24] Karnati S Seimetz M Kleefeldt F Sonawane A Madhusudhan T Bachhuka A Chronic obstructive pulmonary disease and the cardiovascular system: vascular repair and regeneration as a therapeutic target. *Front Cardiovasc Med.* (2021) 8:649512. 10.3389/fcvm.2021.649512 33912600 PMC8072123

[25] Wang Y Xu J Meng Y Adcock IM Yao X. Role of inflammatory cells in airway remodeling in COPD. *Int J Chron Obstruct Pulmon Dis.* (2018) 13:3341–8. 10.2147/COPD.S176122 30349237 PMC6190811

[26] Rabe KF Hurst JR Suissa S. Cardiovascular disease and COPD: dangerous liaisons? *Eur Respir Rev.* (2018) 27:180057. 10.1183/16000617.0057-2018 30282634 PMC9488649

[27] Hillas G Perlikos F Tsiligianni I Tzanakis N. Managing comorbidities in COPD. *Int J Chron Obstruct Pulmon Dis.* (2015) 10:95–109. 10.2147/COPD.S54473 25609943 PMC4293292

[28] Kunisaki KM Dransfield MT Anderson JA Brook RD Calverley PMA Celli BR Exacerbations of chronic obstructive pulmonary disease and cardiac events. a post hoc cohort analysis from the SUMMIT randomized clinical trial. *Am J Respir Crit Care Med.* (2018) 198:51–7. 10.1164/rccm 29442524 PMC6913068

[29] Pinto-Plata VM Livnat G Girish M Cabral H Masdin P Linacre P Systemic cytokines, clinical and physiological changes in patients hospitalized for exacerbation of COPD. *Chest.* (2007) 131:37–43. 10.1378/chest.06-0668 17218554

[30] Chan SMH Selemidis S Bozinovski S Vlahos R. Pathobiological mechanisms underlying metabolic syndrome (MetS) in chronic obstructive pulmonary disease (COPD): clinical significance and therapeutic strategies. *Pharmacol Ther.* (2019) 198:160–88. 10.1016/j.pharmthera.2019.02.013 30822464 PMC7112632

[31] Jin JK Blackwood EA Azizi K Thuerauf DJ Fahem AG Hofmann C ATF6 decreases myocardial ischemia/reperfusion damage and links er stress and oxidative stress signaling pathways in the heart. *Circ Res.* (2017) 120:862–75. 10.1161/CIRCRESAHA.116.310266 27932512 PMC5336510

[32] Akila, D’souza B Vishwanath P D’souza V. Oxidative injury and antioxidants in coronary artery bypass graft surgery: off-pump CABG significantly reduces oxidative stress. *Clin Chim Acta.* (2007) 375:147–52. 10.1016/j.cca.2006.07.001 16904092

[33] Sawyer DB Colucci WS. Mitochondrial oxidative stress in heart failure: “oxygen wastage” revisited. *Circ Res.* (2000) 86:119–20. 10.1161/01.res.86.2.119 10666404

[34] Heymes C Bendall JK Ratajczak P Cave AC Samuel JL Hasenfuss G Increased myocardial NADPH oxidase activity in human heart failure. *J Am Coll Cardiol.* (2003) 41:2164–71. 10.1016/s0735-1097(03)00471-6 12821241

[35] Kiyuna LA Albuquerque RPE Chen CH Mochly-Rosen D Ferreira JCB. Targeting mitochondrial dysfunction and oxidative stress in heart failure: challenges and opportunities. *Free Radic Biol Med.* (2018) 129:155–68. 10.1016/j.freeradbiomed.2018.09.019 30227272 PMC6309415

[36] Scicchitano P Cortese F Gesualdo M De Palo M Massari F Giordano P The role of endothelial dysfunction and oxidative stress in cerebrovascular diseases. *Free Radic Res.* (2019) 53:579–95. 10.1080/10715762.2019.1620939 31106620

[37] Drummond GR Selemidis S Griendling KK Sobey CG. Combating oxidative stress in vascular disease: NADPH oxidases as therapeutic targets. *Nat Rev Drug Discov.* (2011) 10:453–71. 10.1038/nrd3403 21629295 PMC3361719

[38] Samman Tahhan A Sandesara PB Hayek SS Alkhoder A Chivukula K Hammadah M Association between oxidative stress and atrial fibrillation. *Heart Rhythm.* (2017) 14:1849–55. 10.1016/j.hrthm.2017.07.028 28757307 PMC5817893

[39] Yoo S Aistrup G Shiferaw Y Ng J Mohler PJ Hund TJ Oxidative stress creates a unique, CaMKII-mediated substrate for atrial fibrillation in heart failure. *JCI Insight.* (2018) 3:e120728. 10.1172/jci.insight.120728 30385719 PMC6238754

[40] Ren X Wang X Yuan M Tian C Li H Yang X Mechanisms and treatments of oxidative stress in atrial fibrillation. *Curr Pharm Des.* (2018) 24:3062–71. 10.2174/1381612824666180903144042 30179130

[41] Guzik TJ Touyz RM. Oxidative stress, inflammation, and vascular aging in hypertension. *Hypertension.* (2017) 70:660–7. 10.1161/HYPERTENSIONAHA.117.07802 28784646

[42] Ahmad KA Yuan Yuan D Nawaz W Ze H Zhuo CX Talal B Antioxidant therapy for management of oxidative stress induced hypertension. *Free Radic Res.* (2017) 51:428–38. 10.1080/10715762.2017.1322205 28427291

[43] Rodrigo R Prat H Passalacqua W Araya J Guichard C Bächler JP. Relationship between oxidative stress and essential hypertension. *Hypertens Res.* (2007) 30:1159–67. 10.1291/hypres.30.1159 18344620

[44] Blankenberg S Rupprecht HJ Bickel C Torzewski M Hafner G Tiret L Glutathione peroxidase 1 activity and cardiovascular events in patients with coronary artery disease. *N Engl J Med.* (2003) 349:1605–13. 10.1056/NEJMoa030535 14573732

[45] van der Pol A van Gilst WH Voors AA van der Meer P. Treating oxidative stress in heart failure: past, present and future. *Eur J Heart Fail.* (2019) 21:425–35. 10.1002/ejhf.1320 30338885 PMC6607515

[46] Panda P Verma HK Lakkakula S Merchant N Kadir F Rahman S. Biomarkers of oxidative stress tethered to cardiovascular diseases. *Oxid Med Cell Longev.* (2022) 2022:9154295. 10.1155/2022/9154295 35783193 PMC9249518

[47] Dubois-Deruy E Peugnet V Turkieh A Pinet F. Oxidative stress in cardiovascular diseases. *Antioxidants.* (2020) 9:864. 10.3390/antiox9090864 32937950 PMC7554855

[48] Dobrev D Dudley SC. Oxidative stress: a bystander or a causal contributor to atrial remodelling and fibrillation? *Cardiovasc Res.* (2021) 117:2291–3. 10.1093/cvr/cvab124 33822005 PMC8479799

[49] Adameova A Shah AK Dhalla NS. Role of oxidative stress in the genesis of ventricular arrhythmias. *Int J Mol Sci.* (2020) 21:4200. 10.3390/ijms21124200 32545595 PMC7349053

[50] Rodrigues SO Cunha CMCD Soares GMV Silva PL Silva AR Gonçalves-de-Albuquerque CF. Mechanisms, pathophysiology and currently proposed treatments of chronic obstructive pulmonary disease. *Pharmaceuticals.* (2021) 14:979. 10.3390/ph14100979 34681202 PMC8539950

[51] Rahman I. Pharmacological antioxidant strategies as therapeutic interventions for COPD. *Biochim Biophys Acta.* (2012) 1822:714–28. 10.1016/j.bbadis.2011.11.004 22101076 PMC3295924

[52] Anthonisen NR Connett JE Kiley JP Altose MD Bailey WC Buist AS Effects of smoking intervention and the use of an inhaled anticholinergic bronchodilator on the rate of decline of FEV1. The lung health study. *JAMA.* (1994) 272:1497–505. 7966841

[53] Taniguchi A Tsuge M Miyahara N Tsukahara H. Reactive oxygen species and antioxidative defense in chronic obstructive pulmonary disease. *Antioxidants.* (2021) 10:1537. 10.3390/antiox10101537 34679673 PMC8533053

[54] Theodorakopoulou MP Schoina M Sarafidis P. Assessment of endothelial and microvascular function in CKD: older and newer techniques, associated risk factors, and relations with outcomes. *Am J Nephrol.* (2020) 51:931–49. 10.1159/000512263 33311014

[55] Ismaeel A Brumberg RS Kirk JS Papoutsi E Farmer PJ Bohannon WT Oxidative stress and arterial dysfunction in peripheral artery disease. *Antioxidants.* (2018) 7:145. 10.3390/antiox7100145 30347720 PMC6210426

[56] Husain K Hernandez W Ansari RA Ferder L. Inflammation, oxidative stress and renin angiotensin system in atherosclerosis. *World J Biol Chem.* (2015) 6:209–17. 10.4331/wjbc.v6.i3.209 26322175 PMC4549761

[57] Fimognari FL Scarlata S Conte ME Incalzi RA. Mechanisms of atherothrombosis in chronic obstructive pulmonary disease. *Int J Chron Obstruct Pulmon Dis.* (2008) 3:89–96. 10.2147/copd.s1401 18488431 PMC2528208

[58] Endemann DH Schiffrin EL. Endothelial dysfunction. *J Am Soc Nephrol.* (2004) 15:1983–92. 10.1097/01.ASN.0000132474.50966.DA 15284284

[59] Sarafidis PA Bakris GL. Review: insulin and endothelin: an interplay contributing to hypertension development? *J Clin Endocrinol Metab.* (2007) 92:379–85. 10.1210/jc.2006-1819 17118997

[60] Gimbrone Michael A García-Cardeña G. Endothelial cell dysfunction and the pathobiology of atherosclerosis. *Circ Res.* (2016) 118:620–36. 10.1161/CIRCRESAHA.115.306301 26892962 PMC4762052

[61] Peinado VI Barbera JA Ramirez J Gomez FP Roca J Jover L Endothelial dysfunction in pulmonary arteries of patients with mild COPD. *Am J Physiol.* (1998) 274:L908–13. 10.1152/ajplung.1998.274.6.L908 9609729

[62] Ambrosino P Lupoli R Iervolino S De Felice A Pappone N Storino A Clinical assessment of endothelial function in patients with chronic obstructive pulmonary disease: a systematic review with meta-analysis. *Intern Emerg Med.* (2017) 12:877–85. 10.1007/s11739-017-1690-0 28593450

[63] Vaes AW Spruit MA Theunis J Goswami N Vanfleteren LE Franssen FME Endothelial function in patients with chronic obstructive pulmonary disease: a systematic review of studies using flow mediated dilatation. *Expert Rev Respir Med.* (2017) 11:1021–31. 10.1080/17476348.2017.1389277 28978239

[64] Theodorakopoulou MP Alexandrou ME Bakaloudi DR Pitsiou G Stanopoulos I Kontakiotis T Endothelial dysfunction in COPD: a systematic review and meta-analysis of studies using different functional assessment methods. *ERJ Open Res.* (2021) 7:00983–2020. 10.1183/23120541.00983-2020 34195258 PMC8236757

[65] Polverino F Celli BR Owen CA. COPD as an endothelial disorder: endothelial injury linking lesions in the lungs and other organs? (2017 Grover Conference Series). *Pulm Circ.* (2018) 8:2045894018758528. 10.1177/2045894018758528 29468936 PMC5826015

[66] Urban MH Ay L Funk GC Burghuber OC Eickhoff P Wolzt M Insulin resistance may contribute to vascular dysfunction in patients with chronic obstructive pulmonary disease. *Wien Klin Wochenschr.* (2014) 126:106–12. 10.1007/s00508-013-0478-0 24343042

[67] Brassington K Selemidis S Bozinovski S Vlahos R. Chronic obstructive pulmonary disease and atherosclerosis: common mechanisms and novel therapeutics. *Clin Sci.* (2022) 136:405–23. 10.1042/CS20210835 35319068 PMC8968302

[68] Li H Cybulsky MI Gimbrone MA Jr Libby P. An atherogenic diet rapidly induces VCAM-1, a cytokine-regulatable mononuclear leukocyte adhesion molecule, in rabbit aortic endothelium. *Arterioscler Thromb.* (1993) 13:197–204. 10.1161/01.atv.13.2.197 7678986

[69] de Moraes MR da Costa AC Corrêa Kde S Junqueira-Kipnis AP Rabahi MF. Interleukin-6 and interleukin-8 blood levels’ poor association with the severity and clinical profile of ex-smokers with COPD. *Int J Chron Obstruct Pulmon Dis.* (2014) 9:735–43. 10.2147/COPD.S64135 25114519 PMC4122580

[70] Basuroy S Bhattacharya S Leffler CW Parfenova H. Nox4 NADPH oxidase mediates oxidative stress and apoptosis caused by TNF-alpha in cerebral vascular endothelial cells. *Am J Physiol Cell Physiol.* (2009) 296:C422–32. 10.1152/ajpcell.00381.2008 19118162 PMC2660262

[71] Förstermann U. Nitric oxide and oxidative stress in vascular disease. *Pflugers Arch.* (2010) 459:923–39. 20306272 10.1007/s00424-010-0808-2

[72] Bhatt SP Dransfield MT. Chronic obstructive pulmonary disease and cardiovascular disease. *Transl Res.* (2013) 162:237–51. 10.1016/j.trsl.2013.05.001 23727296

[73] Abe H Semba H Takeda N. The roles of hypoxia signaling in the pathogenesis of cardiovascular diseases. *J Atheroscler Thromb.* (2017) 24:884–94. 10.5551/jat.RV17009 28757538 PMC5587513

[74] Morgan AD Zakeri R Quint JK. Defining the relationship between COPD and CVD: what are the implications for clinical practice? *Ther Adv Respir Dis.* (2018) 12:1753465817750524. 10.1177/1753465817750524 29355081 PMC5937157

[75] Crisan L Wong N Sin DD Lee HM. Karma of cardiovascular disease risk factors for prevention and management of major cardiovascular events in the context of acute exacerbations of chronic obstructive pulmonary disease. *Front Cardiovasc Med.* (2019) 25:79. 10.3389/fcvm.2019.00079 31294030 PMC6603127

[76] Trinkmann F Saur J Borggrefe M Akin I. Cardiovascular comorbidities in chronic obstructive pulmonary disease (COPD)-Current considerations for clinical practice. *J Clin Med.* (2019) 8:69. 10.3390/jcm8010069 30634565 PMC6352261

[77] Bernardo I Passey S Seow HJ Bozinovski S Vlahos R. Targeting NADPH oxidase-2 reduces cigarette smoke-induced lung inflammation and skeletal muscle wasting in mice. *Eur Respir Soc.* (2018) 52:OA1945.

[78] Funamoto M Sunagawa Y Katanasaka Y Miyazaki Y Imaizumi A Kakeya H. Highly absorptive curcumin reduces serum atherosclerotic low-density lipoprotein levels in patients with mild COPD. *Int J Chron Obstruct Pulmon Dis.* (2016) 11:2029–34. 10.2147/COPD.S104490 27616885 PMC5008445

[79] Axson EL Bottle A Cowie MR Quint JK. Relationship between heart failure and the risk of acute exacerbation of COPD. *Thorax.* (2021) 76:807–14. 10.1136/thoraxjnl-2020-216390 33927022 PMC8311079

[80] Su VY Yang YH Perng DW Tsai YH Chou KT Su KC. Real-world effectiveness of medications on survival in patients with COPD-heart failure overlap. *Aging.* (2019) 11:3650–67. 10.18632/aging.102004 31175265 PMC6594806

[81] Ponikowski P Voors AA Anker SD Bueno H Cleland JGF Coats AJS 2016 ESC Guidelines for the diagnosis and treatment of acute and chronic heart failure. *Eur J Heart.* (2016) 37:2129–200. 10.1093/eurheartj/ehw128 27206819

[82] Hawkins NM Wang D Petrie MC Pfeffer MA Swedberg K Granger CB Baseline characteristics and outcomes of patients with heart failure receiving bronchodilators in the CHARM programme. *Eur J Heart Fail.* (2010) 12:557–65. 10.1093/eurjhf/hfq040 20356870

[83] Jabbour A Macdonald PS Keogh AM Kotlyar E Mellemkjaer S Coleman CF Differences between beta-blockers in patients with chronic heart failure and chronic obstructive pulmonary disease: a randomized crossover trial. *J Am Coll Cardiol.* (2010) 55:1780–7. 10.1016/j.jacc.2010.01.024 20413026

[84] Li XF Mao YM. Beta-blockers in COPD: a systematic review based on recent research. *Life Sci.* (2020) 252:117649. 10.1016/j.lfs.2020.117649 32275936

[85] Ke Y Xu D Li M Wu Z Huang Y. Effects of bisoprolol in combination with trimetazidine on the treatment of heart failure and concomitant chronic obstructive pulmonary disease. *Pak J Med Sci.* (2016) 32:1208–12. 10.12669/pjms.325.10850 27882023 PMC5103135

[86] Dransfield MT Voelker H Bhatt SP Brenner K Casaburi R Come CE Metoprolol for the prevention of acute exacerbations of COPD. *N Engl J Med.* (2019) 381:2304–14. 10.1056/NEJMoa1908142 31633896 PMC7416529

[87] Kubota Y Tay WT Teng TK Asai K Noda T Kusano K Impact of beta-blocker use on the long-term outcomes of heart failure patients with chronic obstructive pulmonary disease. *ESC Heart Fail.* (2021) 8:3791–9. 10.1002/ehf2.13489 34189870 PMC8497364

[88] Yang YL Xiang ZJ Yang JH Wang WJ Xu ZC Xiang RL. Association of β-blocker use with survival and pulmonary function in patients with chronic obstructive pulmonary and cardiovascular disease: a systematic review and meta-analysis. *Eur Heart J.* (2020) 41:4415–22. 10.1093/eurheartj/ehaa793 33211823 PMC7752251

[89] Noseda G Darioli R Keller U Mordasini R Shokry A Schaffhauser B Prüfung der wirksamkeit und verträglichkeit von atorvastatin bei hyperlipidämie unter praxisbedingungen (SWITCH-Studie) [Evaluating the efficacy and tolerance of atorvastatin in hyperlipidemia in general practice (SWITCH Study)]. *Schweiz Med Wochenschr.* (2000) 130:889–95. 10897490

[90] de Souza Zago VH Tanus-Santos JE Gardin Danelon MR Parra ES Alexandre F Vieira IC Chemical modification of high density lipoprotein subfractions - HDL2 and HDL3 - after use of atorvastatin. *Int J Clin Pharmacol Ther.* (2014) 52:277–83. 10.5414/CP201742 24548977

[91] Bargon J. Statine: stumpfes Schwert gegen COPD-Exazerbationen. *MMW Fortschr Med.* (2020) 162:26–7. 10.1007/s15006-020-0604-7 32578103

[92] Lin CM Yang TM Yang YH Tsai YH Lee CP Chen PC Statin use and the risk of subsequent hospitalized exacerbations in COPD patients with frequent exacerbations. *Int J Chron Obstruct Pulmon Dis.* (2020) 15:289–99. 10.2147/COPD.S229047 32103928 PMC7020922

[93] Neukamm A Høiseth AD Einvik G Lehmann S Hagve TA Søyseth V Rosuvastatin treatment in stable chronic obstructive pulmonary disease (RODEO): a randomized controlled trial. *J Intern Med.* (2015) 278:59–67. 10.1111/joim.12337 25495178

[94] Thomson NC. Clinical studies of statins in asthma and COPD. *Curr Mol Pharmacol.* (2017) 10:60–71. 10.2174/1874467209666160112125911 26758945

[95] Criner GJ Connett JE Aaron SD Albert RK Bailey WC Casaburi R Simvastatin for the prevention of exacerbations in moderate-to-severe COPD. *N Engl J Med.* (2014) 370:2201–10. 10.1056/NEJMoa1403086 24836125 PMC4375247

[96] Yayan J Bald M Franke KJ. No independent influence of statins on the chronic obstructive pulmonary disease exacerbation rate: a cohort observation study over 10 years. *Int J Gen Med.* (2021) 14:2883–92. 10.2147/IJGM.S309647 34234518 PMC8254092

[97] Walsh A Perrem L Khashan AS Henry MT Ni Chroinin M. Statins versus placebo for people with chronic obstructive pulmonary disease. *Cochrane Database Syst Rev.* (2019) 7:CD011959. 10.1002/14651858.CD011959 PMC6699658 31425628

[98] Wang MT Lo YW Tsai CL Chang LC Malone DC Chu CL Statin use and risk of COPD exacerbation requiring hospitalization. *Am J Med* (2013) 126:598–606.e2. 10.1016/j.amjmed.2013.01.036 23684060

[99] Huang CC Chan WL Chen YC Chen TJ Chou KT Lin SJ Statin use and hospitalization in patients with chronic obstructive pulmonary disease: a nationwide population-based cohort study in Taiwan. *Clin Ther.* (2011) 33:1365–70. 10.1016/j.clinthera.2011.08.010 21962452

[100] Blamoun AI Batty GN DeBari VA Rashid AO Sheikh M Khan MA. Statins may reduce episodes of exacerbation and the requirement for intubation in patients with COPD: evidence from a retrospective cohort study. *Int J Clin Pract.* (2008) 62:1373–8. 10.1111/j.1742-1241.2008.01731.x 18422598

[101] Ingebrigtsen TS Marott JL Nordestgaard BG Lange P Hallas J Vestbo J. Statin use and exacerbations in individuals with chronic obstructive pulmonary disease. *Thorax.* (2015) 70:33–40. 10.1136/thoraxjnl-2014-205795 25349333

[102] Wise RA Holbrook JT Brown RH Criner GJ Dransfield MT Han MK Losartan effects on emphysema progression randomized clinical trial. *Chronic Obstr Pulm Dis.* (2021) 8:414–26. 10.15326/jcopdf.2021.0210 34339598 PMC8686851

[103] Wu WT Chen C-Y. Protective effect of statins on pulmonary hypertension in chronic obstructive pulmonary disease patients: a nationwide retrospective, matched cohort study. *Sci Rep.* (2020) 10:3104. 10.1038/s41598-020-59828-0 32080265 PMC7033169

[104] Shrikrishna D Astin R Kemp PR Hopkinson NS. Renin-angiotensin system blockade: a novel therapeutic approach in chronic obstructive pulmonary disease. *Clin Sci.* (2012) 123:487–98. 10.1042/CS20120081 22757959

[105] Ehteshami-Afshar S Mooney L Dewan P Desai AS Lang NN Lefkowitz MP Clinical characteristics and outcomes of patients with heart failure with reduced ejection fraction and chronic obstructive pulmonary disease: insights from PARADIGM-HF. *J Am Heart Assoc.* (2021) 10:e019238. 10.1161/JAHA.120.019238 33522249 PMC7955331

[106] Andreas S Herrmann-Lingen C Raupach T Lüthje L Fabricius JA Hruska N Angiotensin II blockers in obstructive pulmonary disease: a randomised controlled trial. *Eur Respir J.* (2006) 27:972–9. 10.1183/09031936.06.00098105 16446313

[107] Parikh MA Aaron CP Hoffman EA Schwartz JE Madrigano J Austin JHM Angiotensin-converting inhibitors and angiotensin II receptor blockers and longitudinal change in percent emphysema on computed tomography. The Multi-Ethnic Study of Atherosclerosis lung study. *Ann Am Thorac Soc.* (2017) 14:649–58. 10.1513/AnnalsATS.201604-317OC 28207279 PMC5427735

[108] Chandy D Aronow WS Banach M. Current perspectives on treatment of hypertensive patients with chronic obstructive pulmonary disease. *Integr Blood Press Control.* (2013) 6:101–9. 10.2147/IBPC.S33982 23901294 PMC3724277

[109] Lai CC Wang YH Wang CY Wang HC Yu CJ Chen L. Comparative effects of angiotensin-converting enzyme inhibitors and angiotensin II receptor blockers on the risk of pneumonia and severe exacerbations in patients with COPD. *Int J Chron Obstruct Pulmon Dis.* (2018) 13:867–74. 10.2147/COPD.S158634 29563786 PMC5846309

[110] Harrison MT Short P Williamson PA Singanayagam A Chalmers JD Schembri S. Thrombocytosis is associated with increased short and long term mortality after exacerbation of chronic obstructive pulmonary disease: a role for antiplatelet therapy? *Thorax.* (2014) 69:609–15. 10.1136/thoraxjnl-2013-203996 24743560

[111] Maclay JD McAllister DA Johnston S Raftis J McGuinnes C Deans A Increased platelet activation in patients with stable and acute exacerbation of COPD. *Thorax.* (2011) 66:769–74. 10.1136/thx.2010.157529 21507906

[112] Kunadian V Wilson N Stocken DD Ali H McColl E Burns G Antiplatelet therapy in the primary prevention of cardiovascular disease in patients with chronic obstructive pulmonary disease: a randomised controlled proof-of-concept trial. *ERJ Open Res.* (2019) 5:00110–2019. 10.1183/23120541.00110-2019 PMC6680071 31403053

[113] Pavasini R Biscaglia S d’Ascenzo F Del Franco A Contoli M Zaraket F Antiplatelet treatment reduces all-cause mortality in COPD patients: a systematic review and meta-analysis. *COPD.* (2015) 2555:1–6. 10.3109/15412555.2015.1099620 26678708

[114] Ekström MP Hermansson AB Ström KE. Effects of cardiovascular drugs on mortality in severe chronic obstructive pulmonary disease. *Am J Respir Crit Care Med.* (2013) 187:715–20. 10.1164/rccm.201208-1565OC 23328521

[115] Balbirsingh V Mohammed AS Turner AM Newnham M. Cardiovascular disease in chronic obstructive pulmonary disease: a narrative review. *Thorax.* (2022) 30:thoraxjnl–2021–218333. 10.1136/thoraxjnl-2021-218333 35772939

[116] Andell P James SK Cannon CP Cyr DD Himmelmann A Husted S. Ticagrelor versus clopidogrel in patients with acute coronary syndromes and chronic obstructive pulmonary disease: an analysis from the platelet inhibition and patient outcomes (PLATO) trial. *J Am Heart Assoc.* (2015) 4:e002490. 10.1161/JAHA.115.002490 26452988 PMC4845124

[117] Herrin MA Feemster LC Crothers K Uman JE Bryson CL Au DH. Combination antihypertensive therapy among patients with COPD. *Chest.* (2013) 143:1312–20. 10.1378/chest.12-1770 23287970

[118] Vozoris NT Wang X Austin PC O’Donnell DE Aaron SD To TM. Incident diuretic drug use and adverse respiratory events among older adults with chronic obstructive pulmonary disease. *Br J Clin Pharmacol.* (2018) 84:579–89. 10.1111/bcp.13465 29139564 PMC5809361

[119] Èelutkienë J Balèiûnas M Kabluèko D Vaitkevièiûtë L Blašèiuk J Danila E. Challenges of treating acute heart failure in patients with chronic obstructive pulmonary disease. *Card Fail Rev.* (2017) 3:56–61. 10.15420/cfr.2016:23:2 28785477 PMC5494158

[120] Brijker F Heijdra YF van den Elshout FJ Folgering HT. Discontinuation of furosemide decreases PaCO(2) in patients with COPD. *Chest.* (2002) 121:377–82. 10.1378/chest.121.2.377 11834646

[121] Ellison DH Felker GM. Diuretic treatment in heart failure. *N Engl J Med.* (2017) 377:1964–75. 10.1056/NEJMra1703100 29141174 PMC5811193

[122] Khalid K Padda J Komissarov A Colaco LB Padda S Khan AS The coexistence of chronic obstructive pulmonary disease and heart failure. *Cureus.* (2021) 13:e17387. 10.7759/cureus.17387 34584797 PMC8457262

[123] Bruno CM Valenti M. Acid-base disorders in patients with chronic obstructive pulmonary disease: a pathophysiological review. *J Biomed Biotechnol.* (2012) 2012:915150. 10.1155/2012/915150 22500110 PMC3303884

